# Supplementary material for: Performance in a novel environment subject to ghost competition
Source: PeerJ. 2020 Apr 29;8:e8931. doi: 10.7717/peerj.8931 (PMC7195835; doi:10.7717/peerj.8931)
Supplement: Supplemental Information 1 [file peerj-08-8931-s001.docx]

**SUPPLEMENTARY INFORMATION**

Bisschop, K., Mortier, F., Bonte, D, Etienne, R. S., Performance in a novel environment subject to ghost competition, PeerJ

**Figure S1: Comparison of fecundity measured after ten months between replicates.**

The different replicates are given on the x axis and the fecundity (number of eggs after six days) on the y-axis. The coloured points indicate the real measurements. The upper plots are measurements from *T. urticae* on bean with A) the treatment with *T. ludeni* and B) the treatment without *T. ludeni*. The lower plots are measurements from *T. urticae* on cucumber with C) the treatment with *T. ludeni* and D) the treatment without *T. ludeni*.

**Table S1: Overview of the number of samples.**

Summary given per replicate (1 – 8), treatment (T. urticae with and without T. ludeni, and the control population on bean), and plant species for fecundity assessment (bean, B, and cucumber, C) in time (2 – 10 months) for T. urticae. The maximum number per grid is five. Lower numbers indicate adult females that died unnaturally, for instance by drowning before the fecundity assessment. After four months, the cucumber plants did not grow well enough for the fecundity test, therefore we were not able to test fecundity for replicates within that experimental block.

|  |  | 2 months | | 4 months | | 6 months | | 8 months | | 10 months | |
| --- | --- | --- | --- | --- | --- | --- | --- | --- | --- | --- | --- |
|  | replicate | B | C | B | C | B | C | B | C | B | C |
| With *T. ludeni* | 1 | 5 | 5 | 5 |  | 2 | 2 | 5 | 5 | 4 | 4 |
|  | 2 | 5 | 5 | 5 |  | 5 | 5 | 4 | 4 | 5 | 5 |
|  | 3 | 5 | 5 | 4 |  | 5 | 5 | 5 | 5 | 5 | 5 |
|  | 4 | 3 | 3 | 5 |  | 4 | 4 | 5 | 5 | 5 | 5 |
|  | 5 | 5 | 5 | 5 | 4 | 4 | 4 | 4 | 5 | 3 | 3 |
|  | 6 | 5 | 4 | 4 | 4 | 5 | 4 | 5 | 5 | 2 | 2 |
|  | 7 | 5 | 5 | 5 | 5 | 5 | 5 | 4 | 4 | 0 | 1 |
|  | 8 | 3 | 4 | 5 | 4 | 4 | 4 | 5 | 4 | 3 | 3 |
|  | SUM | 36 | 36 | 38 | 17 | 34 | 33 | 37 | 37 | 27 | 28 |
| Without *T. ludeni* | 1 | 4 | 4 | 5 |  | 5 | 5 | 5 | 5 | 5 | 5 |
|  | 2 | 5 | 5 | 4 | 3 | 4 | 4 | 4 | 3 | 3 | 3 |
|  | 3 | 5 | 5 | 5 |  | 3 | 3 | 4 | 4 | 5 | 5 |
|  | 4 | 4 | 4 | 4 |  | 5 | 5 | 5 | 3 | 5 | 5 |
|  | 5 | 4 | 5 | 2 | 2 | 5 | 5 | 5 | 5 | 4 | 4 |
|  | 6 | 5 | 5 | 5 | 5 | 5 | 5 | 4 | 4 | 5 | 5 |
|  | 7 | 5 | 5 | 4 | 4 | 5 | 5 | 4 | 4 | 2 | 2 |
|  | 8 | 5 | 5 | 4 | 4 | 4 | 4 | 5 | 5 | 4 | 4 |
|  | SUM | 37 | 38 | 33 | 18 | 36 | 36 | 36 | 33 | 33 | 33 |
| control | | 31 | 31 | 35 | 17 | 36 | 35 | 36 | 36 | 30 | 31 |

**Table S2: Model selection.** Overview of the best models based on the lowest AICc with an AICc weight of at least 0.100 (with time as a continuous instead of a categorical variable).

|  | Model | df | LogLik | AICc | Δ AICc | AICc weight |
| --- | --- | --- | --- | --- | --- | --- |
| **Signature of the ghost competitor on performance of *T. urticae***  **Fecundity assessed on bean -** max. model: fecundity ~ time + initial density *T. urticae* + initial density *T. ludeni* + time : init. dens. *Tu* + time : init. dens. *Tl* + (1\|block/island) | | | | | | |
| Time | | 5 | -766.927 | 1544.2 | 0.00 | 0.216 |
| No fixed effects | | 4 | -768.410 | 1545.1 | 0.84 | 0.142 |
| Initial density *T. urticae +* Time | | 6 | -766.448 | 1545.4 | 1.19 | 0.119 |
| **Fecundity assessed on cucumber -** max. model: fecundity ~ time + initial density *T. urticae* + initial density *T. ludeni* + time : init. dens. *Tu* + time : init. dens. *Tl* + (1\|island) | | | | | | |
| Initial density *T. ludeni* | | 4 | -594.802 | 1197.9 | 0.00 | 0.280 |
| Time + init. dens. *Tl* | | 5 | -594.177 | 1198.8 | 0.89 | 0.179 |
| Initial dens. *Tu* + initial dens. *Tl* | | 5 | -594.602 | 1199.6 | 1.74 | 0.117 |
| **Performance of *T. urticae***  maximal model: fecundity ~ treatment (*T. urticae* control/*T. urticae* with comp./*T. urticae* without comp.) * time + (1\|block/island) | | | | | | |
| Treatment | | 6 | -1846.641 | 3705.5 | 0.00 | 0.534 |
| Treatment + time | | 7 | -1846.610 | 3707.5 | 2.00 | 0.196 |
| No fixed effects | | 4 | -1849.890 | 3707.9 | 2.40 | 0.161 |

**Table S3: Chi-square statistics for the maximal models before model selection.** The results for the Wald Chi-square tests are presented for the maximal models (with time as a continuous instead of a categorical variable).

|  | Independent variables | Chisq | Df | Pr(>Chisq) |  |
| --- | --- | --- | --- | --- | --- |
| **Signature of the ghost competitor on performance of *T. urticae*** | | | | | |
| Fecundity on bean | Time | 2.6892 | 1 | 0.1010 |  |
|  | Initial density *T. ludeni* | 1.0589 | 1 | 0.3035 |  |
|  | Initial density *T. urticae* | 1.5216 | 1 | 0.2174 |  |
|  | Time : init. dens. *Tl* | 0.0265 | 1 | 0.8708 |  |
|  | Time : init. dens. *Tu* | 0.7537 | 1 | 0.3853 |  |
| Fecundity on cucumber | Time | 1.1777 | 1 | 0.2778 |  |
|  | Initial density *T. ludeni* | 3.3543 | 1 | 0.0670 | . |
|  | Initial density *T. urticae* | 0.3677 | 1 | 0.5443 |  |
|  | Time : init. dens. *Tl* | 0.7408 | 1 | 0.3894 |  |
|  | Time : init. dens. *Tu* | 1.0420 | 1 | 0.3074 |  |
| **Performance of *T. urticae*** | | | | | |
|  | Treatment | 10.2616 | 2 | 0.0059 | ** |
|  | Time | 0.0596 | 1 | 0.8071 |  |
|  | Treatment : time | 1.4170 | 2 | 0.4924 |  |

**Table S4: Summary of the final GLMM explaining reproductive performance.** The values provided in the table are the raw and untransformed estimates (negative binomial distribution) (with time as a continuous instead of a categorical variable).

|  | Estimate | SE | *z* value | *P* value |  |
| --- | --- | --- | --- | --- | --- |
| **Signature of the ghost competitor on performance of *T. urticae*** | | | | | |
| Fecundity assessed on bean | | | | | |
| (Intercept) | 3.8613 | 0.1291 | 29.91 | <2e-16 | *** |
| Time | -0.0268 | 0.0154 | -1.73 | 0.083 | . |
| Fecundity assessed on cucumber | | | | | |
| (Intercept) | 3.2209 | 0.0883 | 36.47 | <2e-16 | *** |
| Initial density *T. ludeni* | 0.0693 | 0.0298 | 2.33 | 0.0199 | * |
| **Performance of *T. urticae*** | | | | | |
| (Intercept) *T. urticae* without comp. | 3.5241 | 0.0395 | 89.23 | <2e-16 | *** |
| *T. urticae* under comp. | -0.1119 | 0.0515 | -2.17 | 0.0297 | * |
| *T. urticae* control | -0.1605 | 0.0516 | -3.11 | 0.0019 | ** |

**Table S5: Pairwise comparisons adjusted for multiple comparisons (Tukey method)** (with time as a continuous instead of a categorical variable).

| Contrast | Estimate | SE | df | t ratio | p value |  |
| --- | --- | --- | --- | --- | --- | --- |
| **Performance of *T. urticae*** | | | | | |  |
| Investigate local adaptation | | | | | |  |
| *T. urticae* (no comp.) – *T. urticae* (comp.) | 0.1119 | 0.0515 | 453 | 2.174 | 0.0767 | . |
| *T. urticae* (no comp.) – *T. urticae* (control) | 0.1605 | 0.0516 | 453 | 3.110 | 0.0056 | ** |
| *T. urticae* (comp.) – *T. urticae* (control) | 0.0486 | 0.0516 | 453 | 0.941 | 0.6144 |  |

**Table S6: Model selection and Wald χ² test for the influence of total initial density on fecundity.** Overview of the best models based on the lowest AICc with an AICc weight of at least 0.100 (with time as a continuous instead of a categorical variable).

|  | Model | df | LogLik | AICc | Δ AICc | AICc weight |
| --- | --- | --- | --- | --- | --- | --- |
| **Fecundity assessed on bean -** max. model: fecundity ~ time + total initial density + time : total initial density + (1\|block/island) | | | | | | |
| Time | | 5 | -766.927 | 1544.2 | 0.00 | 0.288 |
| Time + total initial density | | 6 | -766.074 | 1544.7 | 0.44 | 0.231 |
| No fixed effects | | 4 | -768.410 | 1545.1 | 0.84 | 0.189 |
| Total initial density | | 5 | -767.371 | 1545.1 | 0.89 | 0.185 |
| Time * total initial density | | 7 | -765.740 | 1546.2 | 1.95 | 0.109 |
| **Fecundity assessed on cucumber -** max. model: fecundity ~ time + total initial density + time : total initial density + (1\|island) | | | | | | |
| No fixed effects | | 3 | -597.445 | 1201.1 | 0.00 | 0.396 |
| Time | | 4 | -596.896 | 1202.1 | 1.01 | 0.239 |
| Total initial density | | 4 | -597.132 | 1202.5 | 1.48 | 0.188 |

|  | Independent variables | | Chisq | | Df | | Pr(>Chisq) | |  |
| --- | --- | --- | --- | --- | --- | --- | --- | --- | --- |
| Fecundity on bean | Time | 2.6437 | | 1 | | 0.1040 | |  | |
|  | Total initial density | 1.6858 | | 1 | | 0.1942 | |  | |
|  | Time : total init. dens. | 0.6696 | | 1 | | 0.4132 | |  | |
| Fecundity on cucumber | Time | 1.0035 | | 1 | | 0.3165 | |  | |
|  | Total initial density | 0.5346 | | 1 | | 0.4647 | |  | |
|  | Time : total init. dens. | 1.3809 | | 1 | | 0.2400 | |  | |

**Table S7:**

**Summary of the full GLMM explaining reproductive performance.** The values provided in the table are the raw and untransformed estimates (negative binomial distribution).

|  | Estimate | SE | *z* value | *P* value |  |
| --- | --- | --- | --- | --- | --- |
| **Signature of the ghost competitor on performance of *T. urticae*** | | | | | |
| Fecundity assessed on bean | | | | | |
| (Intercept) (2 months) | 3.569 | 0.434 | 8.22 | <2e-16 | *** |
| 4 months | -0.456 | 0.565 | -0.81 | 0.419 |  |
| 6 months | 0.331 | 0.617 | 0.54 | 0.592 |  |
| 8 months | -0.291 | 0.571 | -0.51 | 0.610 |  |
| 10 months | -0.102 | 0.607 | -0.17 | 0.867 |  |
| Initial density *T. ludeni* | 0.010 | 0.087 | 0.12 | 0.908 |  |
| Initial density *T. urticae* | 0.013 | 0.032 | 0.43 | 0.669 |  |
| 4 months : initial dens. *Tl* | 0.031 | 0.118 | 0.26 | 0.795 |  |
| 6 months : initial dens. *Tl* | -0.005 | 0.127 | -0.04 | 0.970 |  |
| 8 months : initial dens. *Tl* | 0.101 | 0.118 | 0.85 | 0.394 |  |
| 10 months : initial dens. *Tl* | -0.019 | 0.128 | -0.15 | 0.881 |  |
| 4 months : initial dens. *Tu* | 0.058 | 0.043 | 1.34 | 0.179 |  |
| 6 months : initial dens. *Tu* | -0.031 | 0.046 | -0.68 | 0.494 |  |
| 8 months : initial dens. *Tu* | 0.005 | 0.043 | 0.11 | 0.914 |  |
| 10 months : initial dens. *Tu* | -0.013 | 0.048 | -0.28 | 0.781 |  |
| Fecundity assessed on cucumber | | | | | |
| (Intercept) (2 months) | 3.801 | 0.312 | 12.19 | <2e-16 | *** |
| 4 months | -0.646 | 0.570 | -1.13 | 0.257 |  |
| 6 months | -0.607 | 0.456 | -1.33 | 0.183 |  |
| 8 months | -0.905 | 0.456 | -1.99 | 0.047 | * |
| 10 months | -0.468 | 0.464 | -1.01 | 0.313 |  |
| Initial density *T. ludeni* | 0.009 | 0.064 | 0.14 | 0.891 |  |
| Initial density *T. urticae* | -0.048 | 0.024 | -2.02 | 0.043 | * |
| 4 months : initial dens. *Tl* | 0.034 | 0.112 | 0.31 | 0.758 |  |
| 6 months : initial dens. *Tl* | 0.079 | 0.095 | 0.83 | 0.405 |  |
| 8 months : initial dens. *Tl* | 0.116 | 0.093 | 1.25 | 0.211 |  |
| 10 months : initial dens. *Tl* | 0.049 | 0.100 | 0.49 | 0.627 |  |
| 4 months : initial dens. *Tu* | 0.069 | 0.045 | 1.54 | 0.123 |  |
| 6 months : initial dens. *Tu* | 0.054 | 0.034 | 1.58 | 0.115 |  |
| 8 months : initial dens. *Tu* | 0.055 | 0.035 | 1.58 | 0.115 |  |
| 10 months : initial dens. *Tu* | 0.037 | 0.037 | 1.02 | 0.308 |  |
| **Performance of *T. urticae*** | | | | | |
| (Intercept) (*Tu* without comp. 2 months) | 3.581 | 0.073 | 48.88 | <2e-16 | *** |
| *T. urticae* under comp. | -0.126 | 0.102 | -1.23 | 0.220 |  |
| *T. urticae* control | -0.253 | 0.107 | -2.36 | 0.018 | * |
| 4 months | -0.084 | 0.127 | -0.66 | 0.509 |  |
| 6 months | -0.180 | 0.102 | -1.75 | 0.080 | . |
| 8 months | 0.024 | 0.104 | 0.23 | 0.821 |  |
| 10 months | -0.068 | 0.105 | -0.65 | 0.515 |  |
| *Tu* under comp. : 4 months | 0.036 | 0.181 | 0.20 | 0.841 |  |
| *Tu* control : 4 months | 0.098 | 0.184 | 0.53 | 0.593 |  |
| *Tu* under comp. : 6 months | 0.209 | 0.147 | 1.42 | 0.157 |  |
| *Tu* control : 6 months | 0.199 | 0.150 | 1.32 | 0.186 |  |
| *Tu* under comp. : 8 months | -0.178 | 0.147 | -1.21 | 0.225 |  |
| *Tu* control : 8 months | 0.033 | 0.150 | 0.22 | 0.829 |  |
| *Tu* under comp. : 10 months | 0.021 | 0.153 | 0.14 | 0.892 |  |
| *Tu* control : 10 months | 0.144 | 0.154 | 0.94 | 0.347 |  |

**Table S8: Model selection and Wald χ² test for the influence of initial density of T. ludeni and T. urticae on fecundity for smaller datasets.** Overview of the best models based on the lowest AICc with an AICc weight of at least 0.100.

|  | Model | df | LogLik | AICc | Δ AICc | AICc weight |
| --- | --- | --- | --- | --- | --- | --- |
| **Fecundity assessed on cucumber -** max. model: fecundity ~ time + total initial density + time : total initial density + (1\|island) | | | | | | |
| Dataset without second month | | | | | | |
| Initial density *T. ludeni* | | 4 | -454.065 | 916.5 | 0.00 | 0.427 |
| No fixed effects | | 3 | -456.020 | 918.3 | 1.76 | 0.177 |
| Init. dens. *Tl* + init. dens. *Tu* | | 5 | -453.984 | 918.5 | 2.03 | 0.155 |
| Dataset without second and fourth months | | | | | | |
| Initial density *T. ludeni* | | 4 | -384.272 | 777.0 | 0.00 | 0.347 |
| Time + init. dens. *Tl* | | 6 | -382.424 | 777.8 | 0.80 | 0.233 |
| Init. dens. *Tl* + init. dens. *Tu* | | 5 | -384.258 | 779.2 | 2.19 | 0.116 |
| Dataset without second, fourth, and sixth months | | | | | | |
| Initial density *T. ludeni* | | 4 | -258.737 | 526.1 | 0.00 | 0.264 |
| No fixed effects | | 3 | -260.160 | 526.7 | 0.57 | 0.198 |
| Time + init. dens. *T. ludeni* | | 5 | -258.277 | 527.6 | 1.43 | 0.129 |
| Dataset with only 2 months | | | | | | |
| Initial density *T. urticae* | | 4 | -137.733 | 284.8 | 0.00 | 0.555 |
| No fixed effects | | 3 | -139.973 | 286.7 | 1.94 | 0.210 |
| Init. dens. *Tl* + init. dens. *Tu* | | 5 | -137.719 | 287.4 | 2.68 | 0.145 |
| Dataset with only 4 months | | | | | | |
| No fixed effects | | 3 | -68.995 | 145.8 | 0.00 | 0.724 |
| Initial density *T. urticae* | | 4 | -68.961 | 149.3 | 3.42 | 0.131 |
| Initial density *T. ludeni* | | 4 | -68.988 | 149.3 | 3.47 | 0.127 |
| Dataset with only 6 months | | | | | | |
| No fixed effects | | 3 | -120.969 | 248.8 | 0.00 | 0.425 |
| Initial density *T. ludeni* | | 4 | -119.833 | 249.1 | 0.33 | 0.360 |
| Initial density *T. urticae* | | 4 | -120.925 | 251.3 | 2.51 | 0.121 |
| Dataset with only 8 months | | | | | | |
| No fixed effects | | 3 | -150.995 | 308.7 | 0.00 | 0.437 |
| Initial density *T. ludeni* | | 4 | -149.998 | 309.2 | 0.53 | 0.336 |
| Initial density *T. urticae* | | 4 | -150.887 | 311.0 | 2.31 | 0.138 |
| Dataset with only 10 months | | | | | | |
| No fixed effects | | 3 | -106.506 | 220.0 | 0.00 | 0.560 |
| Initial density *T. ludeni* | | 4 | -106.087 | 221.9 | 1.90 | 0.216 |
| Initial density *T. urticae* | | 4 | -106.323 | 222.4 | 2.37 | 0.171 |

|  | Independent variables | | Chisq | | Df | | Pr(>Chisq) | |  |
| --- | --- | --- | --- | --- | --- | --- | --- | --- | --- |
| Dataset without second month | | | | | | | | | |
| Time | | 3.6174 | | 3 | | 0.3059 | |  | |
| Initial density *T. ludeni* | | 4.7933 | | 1 | | 0.0286 | | * | |
| Initial density *T. urticae* | | 0.0719 | | 1 | | 0.7886 | |  | |
| Time : initial dens. *T. ludeni* | | 0.6502 | | 3 | | 0.8848 | |  | |
| Time : initial dens. *T. urticae* | | 0.4646 | | 3 | | 0.9266 | |  | |
| Dataset without second and fouth months | | | | | | | | | |
| Time | | 3.7859 | | 2 | | 0.1506 | |  | |
| Initial density *T. ludeni* | | 5.0893 | | 1 | | 0.0241 | | * | |
| Initial density *T. urticae* | | 0.0068 | | 1 | | 0.9345 | |  | |
| Time : initial dens. *T. ludeni* | | 0.4413 | | 2 | | 0.8020 | |  | |
| Time : initial dens. *T. urticae* | | 0.2636 | | 2 | | 0.8765 | |  | |
| Dataset without second, fourth, and sixth months | | | | | | | | | |
| Time | | 0.9359 | | 1 | | 0.3333 | |  | |
| Initial density *T. ludeni* | | 2.7217 | | 1 | | 0.0990 | | . | |
| Initial density *T. urticae* | | 0.0033 | | 1 | | 0.9542 | |  | |
| Time : initial dens. *T. ludeni* | | 0.3299 | | 1 | | 0.5657 | |  | |
| Time : initial dens. *T. urticae* | | 0.1679 | | 1 | | 0.6820 | |  | |
| Dataset only 2 months | | | | | | | | | |
| Initial density *T. ludeni* | | 0.0272 | | 1 | | 0.8689 | |  | |
| Initial density *T. urticae* | | 4.7746 | | 1 | | 0.0289 | | * | |
| Dataset with only 4 months | | | | | | | | | |
| Initial density *T. ludeni* | | 0.1576 | | 1 | | 0.6913 | |  | |
| Initial density *T. urticae* | | 0.2113 | | 1 | | 0.6457 | |  | |
| Dataset with only 6 months | | | | | | | | | |
| Initial density *T. ludeni* | | 2.4555 | | 1 | | 0.1171 | |  | |
| Initial density *T. urticae* | | 0.1037 | | 1 | | 0.7474 | |  | |
| Dataset with only 8 months | | | | | | | | | |
| Initial density *T. ludeni* | | 1.9279 | | 1 | | 0.1650 | |  | |
| Initial density *T. urticae* | | 0.0408 | | 1 | | 0.8398 | |  | |
| Dataset with only 10 months | | | | | | | | | |
| Initial density *T. ludeni* | | 0.6661 | | 1 | | 0.4144 | |  | |
| Initial density *T. urticae* | | 0.1812 | | 1 | | 0.6704 | |  | |
